# 

**Published:** 1877-04-20

**Authors:** 


☞All communications relating to the business of THE RECORD, for the year 1877. must be addressed to
DR. R. C. WORD,
Business Manager, Southern Med. Rec.,
Atlanta, Ga.

☞Brief and practical communications are solicited on all subjects pertaining to medicine, also reports of cases in practice.

☞Send money by check, postal order, or registered letter.

☞Write your name, post-office, county and State plainly.

AMERICAN MEDICAL ASSOCIATION.—The twenty-eighth Annual Session will be held in the city of Chicago, Ill., on Tuesday, June 5, 1877, in Farwell Hall, at 11 A.M.

The following Committees are expected to report:

On Influence of Climate on Pulmonary Diseases in Florida—Dr. E. T. Sabal, Fla., Chairman.

On Animal Vaccination—Dr. Henry A. Martin, Mass., Chairman.

On the Inheritance of Syphilis—Dr. J. W. Thompson, Ky., Chairman.

On Prize Essays—Dr. N. S. Davis, Ill., Chairman.

On Necrology—Dr. S. C. Chew, Md., Chairman.

On Catalogue of National Library—Dr. H. C. Wood, Pa., Chairman.

Practice of Medicine, Materia Medica, and Physiology—Dr. P. G. Robinson, St. Louis, Mo., Chairman.

Committee appointed to report to this Section:

On Clinical Observations—Dr. N. S. Davis, Ill., Chairman.

Obstetrics and Diseases of Women and Children—Dr. James P. White, Buffalo, N. Y., Chairman.

Surgery and Anatomy—Dr. Moses Gunn, Chicago, Ill., Secretary.

Medical Jurisprudence, Chemistry, and Psychology—Dr. Eugene Grissom, Raleigh, N. C., Chairman.

State Medicine and Public Hygiene—Dr. Ezra M. Hunt, Metuchen, N. J., Chairman.

W. B. ATKINSON, M.D., Philadelphia,
Permanent Secretary.

COLABORATORS AND CONTRIBUTORS

L. B. Anderson, M.D., Va.
Swan M. Burnett, M.D., D. C
J. M. Bristow M.D., Texas
J. W. Booth, M.D., N C.
W. F. Barr, M.D., Va.
B. W. Corn, M.D., Texas.
John Castor, M.D., Iowa.
E. T. Easley. A.M., M.D., Ark.
A. H. Goelet, M D., N. Y.
E. C. Gehrung, M.D., Mo.
G. D. Hodge, M.D., Ark.
S. M. Hogan, M.D., Ala.
A. McLane Hamilton, M.D., N.Y.
Z. B. Herndon, M.D., Va.
Lindsay Johnson, M.D., Ga.
A. R. Kilpatrick, M.D., Tex.
R. Kells, M.D., Miss.
W. L. Lipscomb, M.D., Miss.
J. M. Lewis, M.D., Miss.
C. H. Lathrop, M.D., Iowa.
A. A. Lyon, M.D., La.
M. Lewis, M.D., Tenn.
G. M. Rivers, M.D., S. C.
A. S. Payne, M.D., Va.
A. M. Whitehead, M.D., O.

